# African meningitis belt pneumococcal disease epidemiology indicates a need for an effective serotype 1 containing vaccine, including for older children and adults

**DOI:** 10.1186/1471-2334-10-22

**Published:** 2010-02-10

**Authors:** Bradford D Gessner, Judith E Mueller, Seydou Yaro

**Affiliations:** 1 Agence de Médecine Préventive, 25-28 Rue du Dr. Roux, Paris, 75728, France; 2 Centre Muraz, BP 390, Bobo-Dioulasso, 01, Burkina Faso

## Abstract

**Background:**

Pneumococcal conjugate vaccine strategies in GAVI-eligible countries are focusing on infant immunization but this strategy may not be optimal in all settings. We aimed to collect all available population based data on pneumococcal meningitis throughout life in the African meningitis belt and then to model overall meningitis risk to help inform vaccine policy.

**Methods:**

After a systematic review of literature published from 1970 through the present, we found robust population-based *Streptococcus pneumoniae *(Sp) meningitis data across age strata for four African meningitis belt countries that included 35 surveillance years spanning from 1970 to 2005. Using these data we modeled disease risk for a hypothetical cohort of 100,000 persons followed throughout life.

**Results:**

Similar to meningococcal meningitis, laboratory-confirmed pneumococcal meningitis was seasonal, occurring primarily in the dry season. The mean annual Sp meningitis incidence rates were 98, 7.8 to 14, and 5.8 to 12 per 100,000 among persons <1, 1 through 19, and 20 to 99 years of age, respectively, which (in the absence of major epidemics) were higher than meningococcal meningitis incidences for persons less than 1 and over 20 years of age. Mean Sp meningitis case fatality ratios (CFR) among hospitalized patients ranged from 36-66% depending on the age group, with CFR exceeding 60% for all age groups beyond 40 years; depending on the age group, Sp meningitis mortality incidences were 2 to 12-fold greater than those for meningococcal meningitis. The lifetime risks of pneumococcal meningitis disease and death were 0.6% (1 in 170) and 0.3% (1 in 304), respectively. The incidences of these outcomes were highest among children age <1 year. However, the cumulative risk was highest among persons age 5 to 59 years who experienced 59% of pneumococcal meningitis outcomes. After age 5 years and depending on the country, 59-79% of meningitis cases were caused by serotype 1.

**Conclusions:**

In the African meningitis belt, Sp is as important a cause of meningitis as *Neisseria meningitidis*, particularly among older children and working age adults. The meningitis belt population needs an effective serotype 1 containing vaccine and policy discussions should consider vaccine use outside of early childhood.

## Background

Pneumococcal vaccine policy discussions for Africa have focused almost exclusively on developing a pneumococcal conjugate vaccine for use in infancy or early childhood [1,2]. The Global Alliance for Vaccines and Immunization (GAVI)-sponsored PneumoADIP has a mission statement limited to accelerating "access to new, lifesaving pneumococcal vaccines for the world's children." (website: http://www.preventpneumo.org/mission.cfm, last accessed June 24, 2008). Fewer discussions have occurred regarding pneumococcal vaccine use among older children and adults and no vaccine strategies have been formulated. This focus on pediatric disease may be misplaced in the African meningitis belt [3], a region of sub-Saharan Africa that is characterized by particularly high and seasonal incidences of bacterial meningitis and annual rainfall between 300 and 1100 mm. For example, data we collected from an exhaustive polymerase chain reaction-based surveillance system in and around Bobo-Dioulasso, Burkina Faso, during 2002-7 suggest that high pneumococcal meningitis burden extends throughout life and is associated with a high case fatality ratio [4-6]. The current report aims to summarize and model data on pneumococcal meningitis burden in the African meningitis belt and to discuss implications for vaccine policy.

## Methods

### Search criteria

We searched PubMed, CINAHL Plus, and the ISI Web of Knowledge (containing SCI-Expanded, SSCI, and A&HCI) databases and the references of retrieved articles. English and French language articles from 1970 to the present were included. A single author (BDG) performed all data abstraction and entered data into an Excel spreadsheet. No validity assessment of retrieved articles was performed.

### Article retrieval

The primary meningitis outcome was the Sp meningitis incidence by age group throughout life in the African meningitis belt. Step one of the search was designed to be sensitive in identifying potential articles. Consequently, search terms were non-specific and included "meningitis" in combination with one of the meningitis belt countries including (alphabetically) Burkina Faso, Chad, Ethiopia, The Gambia, Guinea, Guinea Bissau, Mali, Niger, Senegal, and Sudan plus the northern half of the Central African Republic, Cote d'Ivoire (and Ivory Coast), Ghana, Kenya, Nigeria, Togo, and Uganda. Articles that reported only meningococcal, *Haemophilus influenzae *type b (Hib), or epidemic meningitis in their title had their abstracts reviewed but then uniformly excluded as having no information on pneumococcal disease. This led to identification of 45 articles, including 12 from The Gambia, eight from Ethiopia, five each from Northern Nigeria and Senegal, three each from Mali, Niger and Burkina Faso, two from Cote d'Ivoire, and one each from Chad, northern Ghana, northern Togo, and northern Uganda.

Step two involved reviewing the retrieved articles to identify those that reported age-specific pneumococcal meningitis incidence throughout life. Only five articles were identified that provided this information and thus that were included in the evaluation [4,5,7-9]. Four of these articles [4,5,8,9] were of approximately the same quality in that they determined incidence by conducting hospital and health center surveillance for cases and then divided by the estimated population of the area from which cases derived. In many settings in Africa, though, population estimates may not be accurate. Consequently, for these studies incidence rates should be considered rough approximations. The fifth article from Ghana [7] followed a defined population over time. All of the studies were used to estimate meningitis burden; the two studies from Burkina Faso [4,5] reported overlapping data from the same surveillance site and some of the same time periods and thus data were combined. Each of the articles reported surveillance that spanned all months of the year. However, we could not exclude the possibility that persons with meningitis symptoms were more likely to present or receive an evaluation (i.e., lumbar puncture and laboratory evaluation) during the epidemic meningitis season months.

Each of the articles reported surveillance that spanned all months of the year. However, we could not exclude the possibility that persons with meningitis symptoms were more likely to present to a health care facility and receive an evaluation during the epidemic meningitis season. This could have led to an underestimation of pneumococcal meningitis incidence, including by serotype if some serotypes occurred preferentially during particular seasons. This could have been assessed in part by evaluating seasonality by age and serotype but none of the studies reported this information. The similar magnitude of increase of meningococcal and pneumococcal meningitis during the meningitis season and the known seasonality of meningococcal meningitis suggest that this issue did not affect results to a large degree.

### Model structure

Besides reporting the results from individual studies, we estimated the lifetime risk of pneumococcal disease outcomes. First, an average age-specific annual meningitis incidence rate - weighted for the size of the population under surveillance - was calculated for the four included meningitis belt countries. Annual meningitis mortality incidence rates were calculated as the weighted average of the case fatality ratios multiplied by the annual meningitis incidence rates within each age category. To calculate lifetime meningitis risk, age-specific pneumococcal meningitis and meningitis mortality incidence rates were applied to a hypothetical population of 100,000 persons followed from birth until the 100^th ^birthday (by which point all persons were assumed to have died). Data are presented by age group through age 99 years. Not all studies reported age groups at the same level of detail; where data were missing, the incidence rate for the closest available older age group was used. Because data were sparse particularly for older ages, for all outcomes identical values were used from age 60 through 99 years. Ghana did not report age-specific case fatality ratios and thus was not included in estimation of pneumococcal meningitis mortality incidence rates. All estimates were adjusted for all-cause mortality from all causes based on life tables for the four study countries (website: http://www.who.int/whosis/database/life_tables/life_tables.cfm, last accessed July 2, 2008). This adjustment thus accounts for the relatively low life expectancies found in the analyzed countries.

The four study countries reported meningitis incidence rates only among persons who presented to a hospital or health center and who had a diagnostic workup that led to documentation of meningitis. However, an unknown number of persons have undocumented meningitis because of failure to present, pretreatment with antibiotics, lack of lumbar puncture, or problems with specimen transport or storage. Anecdotal reports from our surveillance site in Burkina Faso and our experience in Senegal, Mali, Togo, and Niger suggest awareness of meningitis and care seeking are relatively high, but the contribution of other factors remains unknown. For example, during the dry epidemic meningitis season months, treatment may be presumptive based on clinical symptoms or documentation of purulent cerebrospinal fluid without confirmation of etiology. Manuscripts included in the current analysis do not indicate any attempts by the study teams to increase access to care or increase the percent of cases with etiological confirmation (indeed, the Niger and Senegal studies were retrospective). However, because data from the meningitis belt on the effect of the factors discussed above do not exist, we did not make an attempt to model the burden of undocumented cases.

The African meningitis belt traditionally has been considered to have abnormally high meningococcal - but not necessarily pneumococcal - disease burden [3]. As a point of comparison, we present data on meningococcal meningitis disease incidence and mortality rates. Niger [8] and Senegal [9] reported these data in the same manuscript while Burkina Faso reported data from the same time period and surveillance site in a separate manuscript [10]. Comparison data were not available for Ghana.

## Results

The four countries reporting meningitis incidence rates throughout life included two major metropolitan areas plus rural areas, involved in aggregate 36 years of follow-up, spanned from 1970 to 2005, and identified 2,242 persons with pneumococcal meningitis (Table 1). At all four sites, pneumococcal meningitis was seasonal with the highest number of cases during the dry season (i.e., the epidemic meningitis season). For three sites, pneumococcal meningitis was highly seasonal with case identification decreasing to near zero during other months. Three studies reported serotype results and among those at least 5 years of age (or 2 years for Senegal) serotype 1 contributed at least 60% of cases for all three sites (Table 2).

**Table 1:** Characteristics of evaluated countries.

Characteristic	Burkina Faso*	Senegal*	Niger*	Ghana*
Surveillance design	Prospective	Retrospective	Retrospective	Prospective

Geographic extent	Three districts	Dakar	Niamey	Northern area

Surveillance population	880,000	950,000	550,000	140,000

Rural/Urban	Both	Urban	Urban	Rural

Study years	2002-2005	1970-1979	1981-1996	1998-2003

Microbiological methods	Culture, antigen detection, PCR	Culture	Culture, antigen detection	Culture, antigen detection

Number of confirmed pneumococcal meningitis cases	249	983	934	76

Percent of cases occurring during dry/epidemic season months of Dec-Apr^†^	75%	49%	63%	69%

Two months with peak number of cases (percent of total)^†^	Jan-Feb (38%)	Feb-Mar (21%)	Feb-Mar (31%)	Dec-Jan (35%)

Relation of pneumococcal to meningococcal seasonal peak	Similar	2 months in advance	1 month in advance	1 to 2 months in advance

13-valent conjugate vaccine coverage of isolated serotypes				

Age <5 years	67%	78% (age<2 yrs)	No serotype data	100%

Age 5+ years	77%	94% (age 2+ yrs)	No serotype data	98%

Reference	4,5	9	8	7

* Human immunodeficiency virus prevalence in pregnant women age 15-24 years in capital city: Burkina Faso 2.3% (2002), Ghana 3.9% (2003), Senegal 1.1% (2003). Adult HIV prevalence in Niger was 1.2% (2003). Website: http://www.unicef.org/publications/files/SOWC_2005_(English).pdf, last accessed October 2, 2008.

† In all studies cases per month were estimated from Figures as specific data were not presented.

**Table 2:** Serogroup/type distribution by age group and country.

Serotype	Burkina Faso		Senegal		Ghana	
	**Age <** **5 years**	**Age ** **5+ years**	**Age <** **2 years**	**Age ** **2+ years**	**Age <** **5 years**	**Age ** **5+ years**

1	3 (17%)	18 (60%)	10 (9%)	90 (63%)	2 (33%)	51 (80%)

2	3 (17%)		15 (14%)	3 (2%)		

3			6 (5%)	8 (6%)	2 (33%)	2 (3%)

5	3 (17%)		23 (21%)	8 (6%)		

6A	5 (28%)		14 (13%)*	13 (9%)*		1 (2%)
			
6B	1 (6%)					

7/7F		1 (3%)				1 (2%)

8						4 (6%)

9			6 (5%)	2 (1%)		

10/10F	1 (6%)					1 (2%)

12A		1 (3%)	9 (8%)*	6 (4%)*		
			
12B		1 (3%)				
			
12F						2 (3%)

14		2 (7%)	5 (5%)	2 (1%)	2 (33%)	1 (2%)

18			4 (4%)	2 (1%)		

19A		1 (3%)	4 (4%)*	4 (3%)*		
			
19F		1 (3%)				

21	1 (6%)					

23			14 (13%)	5 (3%)		

24A	1 (6%)					

25A		2 (7%)				

25F		2 (7%)				

38						1 (2%)

NT		1 (3%)				

Total	18 (100%)	30 (100%)	110 (100%)	143	6 (100%)	64 (100%)

* These cells present summary data on serogroups 6, 12, and 19 as serotype specific data were not available

Age-specific annual laboratory-confirmed pneumococcal meningitis incidence rates were highest among infants (Table 3). Children age 12 to 23 months also may have been at increased risk but only one study reported results specifically for this age group. Following the first two years of life, and at all four study sites, age-specific annual pneumococcal meningitis incidence were consistent and high for all age groups, with weighted means ranging from 6 to 14 per 100,000 per year. Niger, Burkina Faso, and Senegal also reported - from the same study site and period - data for meningitis due to *Neisseria meningitidis*, the etiology typically associated with the African meningitis belt; however, none of the studies included data from years during which major meningococcal meningitis epidemics occurred. Compared to endemic meningococcal meningitis, pneumococcal meningitis incidence rates were higher in infancy, similar in early childhood, lower in later childhood, similar in young adulthood and then substantially higher from age 30 years on. Pneumococcal case fatality ratios were reported by age group for three sites and were uniformly high, ranging from 36% to 66% (Table 4); the fourth country (Ghana) reported an overall pneumococcal meningitis case fatality ratio of 44%. Compared to meningococcal meningitis, pneumococcal case fatality ratios were higher for all age groups and annual mortality incidence rates were higher by a factor of 2 to 18.

**Table 3:** *Streptococcus pneumoniae *(Sp) meningitis incidence rates (per 100,000 per year) by age group and country [4,5,7-9]; *Neisseria meningitidis *meningitis incidences were available from Burkina Faso, Senegal, and Niger and summary means are presented for comparison.

Age (yrs)	Burkina Faso	Senegal	Ghana*	Niger*	Mean^†^ for Sp	Mean^†^ for Nm^‡^
<1	77	95	42	150	98	24

1 to 4	14	17	19	10	14	20

5 to 9	13	4.5	25	6.4	9.1	19
		
10 to 14	10	3.7		6.9	7.8	17

15 to 19	11	2.4	20	9	7.8	11
		
20 to 29	6.1	4.0		4.7	5.8	5.0

30 to 39	14.1	5.2	18	3.2	8.6	3.3
		
40 to 49	19.1	7.8		7.9	12	2.8
			
50 to 59	8.0	11			9.7	3.3
		
60+	8.1	15	25		10	2.8

*Not all studies reported age groups at the detail shown in column 1. The incidences in Ghana for persons age 5 to 19, 15 to 29, and 30 to 59 years and in Niger for persons 40+ years were 25, 20, 18, and 7.9 per 100,000 persons per year, respectively.

^†^Weighted mean based on population under surveillance. Where data were not available from a study site for a specific age group, the next oldest age group was used in the calculation of the weighted mean.

^‡^ Nm = *Neisseria meningitidis*. For comparison, data on Nm are presented for the same surveillance periods and sites except for Ghana, which did not report these data.

**Table 4:** *Streptococcus pneumoniae *(Sp) meningitis case fatality ratios by age group and country [4,5,7-9]; *Neisseria meningitidis *meningitis case fatality ratios were available from Burkina Faso, Senegal, and Niger and summary means are presented for comparison.

**Age (yrs)**	Burkina Faso*	**Senegal**	Niger*	Ghana*	**Mean^†^ Sp CFR** **(mortality)^‡^**	**Mean^†^ Nm^‡^** **CFR (mortality^§^)**
		
<1	52%	55%	58%	44%	53% (53)	12% (2.9)
		
1 to 4	50%	50%	57%		52% (7.4)	17% (3.3)
		
5 to 9	45%	48%	16%		36% (3.6)	10% (1.8)
				
10 to 14		50%	35%		43% (3.5)	10% (1.7)
		
15 to 19	44%	35%	62%		47% (3.5)	9% (1.0)
				
20 to 29		60%	58%		54% (3.1)	16% (0.8)
				
30 to 39		70%	20%		45% (4.2)	24% (0.8)
				
40 to 49		85%	60%		63% (7.9)	21% (0.6)
					
**50 to 59**		78%			61% (5.9)	43% (1.4)
					
60+		93%			66% (6.7)	43% (1.2)

*Not all studies reported age groups at the detail shown in column 1. The CFRs in Burkina Faso for persons age 5 to 14 and 15+ years, in Niger for persons 40+ years, and in Ghana for all ages combined were 45%, 44%, 60%, and 44%, respectively.

^†^Weighted mean based on population under surveillance; Ghana was not included in the weighted means. Where data were not available from a study site for a specific age group, the next oldest age group was used in the calculation of the weighted mean.

^‡^ Nm = *Neisseria meningitidis*. For comparison, data on Nm are presented for the same surveillance periods and sites except for Ghana, which did not report these data.

^§^Mortality rate per 100,000 per year, based on the product of the meningitis incidence rate and the case fatality ratio.

The overall estimated lifetime risk of laboratory-confirmed pneumococcal meningitis was 0.6% (1 in 170 persons) and for laboratory-confirmed pneumococcal meningitis death was 0.3% (1 in 304 persons). Infants age <1 year (i.e., the age group targeted by pneumococcal conjugate vaccine childhood immunization programs) experienced 17% of cases and 16% of deaths, while children age less than 5 years experienced 25% of cases and 24% of deaths. Persons age 60 years or more (i.e., one target age group for pneumococcal polysaccharide vaccine) experienced 16% of cases and 19% of deaths. By contrast, persons age 5 to 59 years of age, who usually are not targeted by vaccination programs, experienced 59% of cases and 57% of deaths.

**Figure 1:**
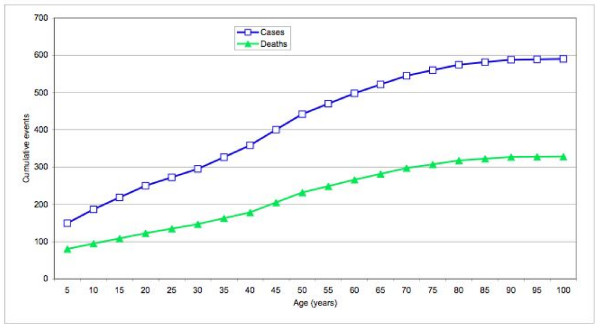
**Cumulative *Streptococcus pneumoniae *meningitis and meningitis death cases**. Among a hypothetical population of 100,000 persons living in the meningitis belt and followed from birth to death, the cumulative number of *Streptococcus pneumoniae *meningitis and meningitis death cases occurring by 5-year age increments.

The cumulative risk of pneumococcal meningitis and meningitis death increased at a relatively constant rate after age 5 years (Figure 1). The number of new cases decreased among older persons despite a modest increase in incidence rates because fewer and fewer people remained alive to experience outcomes. The total number of outcomes was greatest during the first year of life (Table 5). Nevertheless, the number of new cases occurring during each age decade after 20 years was comparable to that during infancy and up to three times greater than that during the age category of 1-9 years.

**Table 5:** Estimates of laboratory-confirmed *Streptococcus pneumoniae *(Sp) meningitis cases and deaths within age groups.

Age group in years	Meningitis cases	Meningitis deaths
<1	98	53

1 to 4	52	27

5 to 9	37	15

10 to 14	32	14

15 to 19	31	14

20 to 29	45	24

30 to 39	64	31

40 to 49	83	53

50 to 59	56	34

60 to 69	47	32

70 to 79	30	20

80 to 89	10	7

90 to 99	2	1

Total	587	326

These estimates were made for a hypothetical population of 100,000 persons in the African meningitis belt followed from birth through age 99 years [4,5,7-9].

## Discussion

### Meningitis

The current analysis showed that for at least the last 35 years, and based on approximately 35 years of surveillance occurring in four countries, pneumococcal meningitis in the meningitis belt has caused a high burden of disease and high mortality in older children and working age adults, primarily during the epidemic meningitis season. Of the estimated 1 in 170 persons that will experience laboratory-confirmed pneumococcal meningitis, many will develop sequelae such as hearing and vision loss, seizure disorder, cerebral palsy, and mental retardation [11], although no specific data exist from the meningitis belt on these sequelae among older children and adults. Beyond laboratory-confirmed cases, an unknown burden of unconfirmed cases exists, since some persons do not present for care or receive a lumbar puncture, others have received pretreatment with antibiotics, and laboratory limitations may prevent etiologic identification. This issue may be accentuated for mortality, since lumbar punctures may be less likely to be performed in critically ill and unstable patients. Consequently, data presented very here should be considered minimum estimates.

Within the limits of our analysis, pneumococcal meningitis burden among older children and working age adults may surpass that for diseases currently targeted for routine immunization in meningitis belt countries. For example, WHO has recommended universal infant Hib vaccination [12], and the meningitis belt in particular has among the world's highest annual incidence rates at 34-60 per 100,000 children aged under 5 years [13]. Almost the entire risk of Hib disease, though, occurs during childhood. By contrast, the high pneumococcal meningitis incidences that occur throughout life result in a higher pneumococcal meningitis risk during ages 5 to 49 years than Hib meningitis during childhood. Beyond the higher cumulative risk of pneumococcal outcomes, disease, disability, and death among working adults may impoverish entire families or extended families, triggering a cascade of adverse health events [14].

Compared to the United States and Europe, where a bimodal meningitis age distribution with peaks in infants and the elderly is observed, pneumococcal meningitis epidemiology in the meningitis belt appears distinctly different, with strong seasonality, predominance of serotype 1 outside of childhood, higher incidences, higher case fatality ratios, and an age distribution with a concentration on older children and working age adults [15-19]. Indigenous persons of North America and Australia have overall pneumococcal disease incidences that are among the world's highest. However, Sp meningitis incidence in these populations is still relatively low compared to incidence rates observed in the African meningitis belt, and other aspects of Sp epidemiology are similar to what is seen in developed country populations [16,20,21]. African countries outside the meningitis belt have, not reported population-based pneumococcal meningitis data across the entire life span. An unpublished study from Kenya [22] reported annual incidence rates among adults for all invasive pneumococcal disease - including bacteremia, which can cause 10-20 fold more invasive disease than meningitis [15] - of 261 and 3.3 per 100,000 among, respectively, persons with and without confirmed human immunodeficiency virus (HIV) infection. The latter rate for all invasive disease is substantially lower than the Sp meningitis incidence seen in the meningitis belt. North-central Uganda, which borders the meningitis belt, reported an annual pneumococcal meningitis incidence among children under age 5 years of 28 to 42 per 100,000 versus 3 to 20 per 100,000 reported in tropical Kampala [23]. These data suggest that the meningitis belt has a unique pneumococcal disease epidemiology even within Africa.

The mechanisms responsible for the Sp epidemiology found in the meningitis belt remain unknown. The observed seasonal pattern indicates some overlap in risk factors with meningococcal meningitis [24] including climatic conditions, concurrent respiratory infections, decreased host immunity and others. Although HIV plays an important role in many African countries [22], meningitis belt countries have relatively low HIV prevalences and the epidemiology of pneumococcal meningitis in the region has been relatively stable since the 1970s when HIV was presumably of little importance. It is unlikely that circulation and transmission of serotype 1 alone explains the observed patterns since Asia, with the world's lowest documented pneumococcal meningitis incidences, reports serotype 1 as the most common cause of meningitis in all age groups [25]. Lastly, meningitis belt countries have a high prevalence of hemoglobinopathies mainly due to hemoglobin S and C [26], and these greatly increase the risk of invasive pneumococcal disease [27]. High hemoglobinopathy prevalence, however, is not unique to the meningitis belt.

### Implications for pneumococcal pneumonia

The African meningitis belt, home to about 350 million people, is characterized by an extraordinarily high incidence of acute bacterial meningitis, occurring mainly during the dry season. No data, however, exist from the meningitis belt on age specific pneumonia incidence, either overall or specifically for pneumococcus. Thus, it remains unknown if the high pneumococcal meningitis disease burden occurs in association with a high pneumococcal pneumonia burden. In industrialized countries, numerous studies have estimated the ratio of bacteremic pneumococcal pneumonia to meningitis [15-21,28,29]. Examination of data from these studies (Additional file 1, Table S1) illustrates that this ratio remains relatively stable across populations (including high incidence populations such as Alaska Native people) and increases sharply with age. If ratios seen in developed countries hold true for meningitis belt countries, it would imply a very high risk of pneumococcal pneumonia (estimated as 1 in every 15 persons using the presented meningitis data) and overall pneumococcal mortality (estimated as 1 in every 62 persons) with a risk even more weighted toward ages outside of early childhood than that seen for meningitis.

The applicability of this ratio for meningitis belt populations, however, is unknown. The only relevant current data point from in or near the meningitis belt is the pneumococcal conjugate vaccine trial (using a serotype 1 containing vaccine) from The Gambia, located just outside the Western edge of the meningitis belt [30]. Based on reported vaccine-preventable disease incidences for various outcomes, the ratio of vaccine-preventable pneumococcal pneumonia (bacteremic and non-bacteremic) to meningitis was 30 to 1 and the ratio of all pneumococcal mortality to pneumococcal meningitis mortality was 8.8 to 1. These figures would imply an even higher risk of Sp pneumonia and mortality than the values reported in the previous paragraph. The Gambia trial, though, is limited by its location outside of the meningitis belt and a study population limited to children age 6 weeks to 2 years. In summary, there is a need for data on pneumococcal pneumonia across age groups specifically for the meningitis belt.

### Limitations

Our study had at least six limitations. Data were not available from countries located in the eastern part of the meningitis belt such as Sudan and Ethiopia, where pneumococcal disease epidemiology in theory may be different. Meningitis incidence estimates from Niger, Burkina Faso, and Senegal were based on dividing case counts by the population data available, which may be imprecise and therefore overestimate or underestimate true incidence rates. We did not find data on the proportion of all persons with pneumococcal meningitis that present for definitive diagnosis either overall or by syndrome or age group. Although serotype 1 appears to have predominated over many years in the meningitis belt, serotype distribution is a dynamic process. Our results are valid for a situation with predominance of serotype 1 in older children and adults and may require revision if the serotype distribution should change. Only Senegal provided mortality data restricted to elderly persons and thus estimates for this group are uncertain. Lastly, a quality assessment of the studies was not conducted and approximations in denominators and inclusion of different time periods could have affected results.

### Implications for vaccine use in the meningitis belt

Existing data indicate that meningitis belt populations need a serotype 1-containing vaccine. The licensed 7-valent pneumococcal conjugate vaccine does not contain serotype 1, may be associated with increases in non-vaccine serotypes [31-34], and thus likely will have little role outside of early childhood in the meningitis belt. Existing 23-valent polysaccharide vaccines could have a role, but these vaccines do not reduce carriage, may be no less expensive than conjugate vaccines, and manufacturers may have little interest in increasing production. Before or concurrent with introduction, the effectiveness of serotype 1 containing vaccine must be confirmed. In The Gambian trial, vaccine was not effective against serotype 1 disease, however the results were based on a total of only six invasive isolates [35]. Other studies - including in The Gambia - have documented robust immunologic response against serotype 1 [35-38] as well as vaccine effectiveness with conjugate [39] or polysaccharide vaccines [40]. The latter finding is encouraging since conjugate vaccines usually elicit more robust immune responses, including for serotype 1 and outside of childhood [41,42]. Nevertheless, questions about serotype 1 conjugate vaccine effectiveness must be resolved before widespread vaccine implementation.

A policy of universal infant pneumococcal vaccination may provide indirect protection to older persons if ongoing Sp carriage and transmission requires young children [43]. If transmission for some serotypes can be maintained solely among older persons, though, infant vaccination will have little impact on the pneumococcal disease burden we describe here. Data on serotype 1 transmission dynamics, including the contribution of different age groups, are lacking in the meningitis belt. Among African populations outside of the meningitis belt, serotype 1 carriage is rarely seen [44-46] but this may indicate a short carriage duration rather than infrequent transmission. During a serogroup A meningococcal epidemic in Burkina Faso, we found among a representative community-based population sample of persons aged 1 to 39 years relatively flat age distributions of overall pneumococcal carriage as well as IgG seroprevalence for serotype 1 similar to other serotypes. Moreover two of three serotype 1 carriage isolates were identified from persons aged greater than 5 years [6].

Most logistical issues associated with delivering pneumococcal vaccine outside of infancy have already been addressed during planning by the GAVI Alliance, WHO, and Unicef for preventive conjugate meningococcal serogroup A vaccine introduction and for the current yellow fever vaccine campaigns in sub-Saharan Africa. For example, one proposed plan for the meningococcal vaccine is to conduct an initial mass vaccination campaign among persons 1 to 29 years of age, followed by continuous routine infant vaccination. Similar mass vaccination campaigns with pneumococcal conjugate vaccine likely would have an immediate and high impact on pneumococcal disease in the region, and possibly higher than that associated with routine infant immunization. Because of longer antibody persistence among individuals older than 1 year, a single dose among persons 1 to 29 years of age probably would have a long-term impact on pneumococcal disease burden. Mass campaigns and routine infant immunization could occur simultaneously, although costs, vaccine availability, and programmatic issues may require a sequential approach. Efforts also should be made to improve and document high vaccination coverage.

## Conclusions

Based on available data for pneumococcal disease epidemiology in the African meningitis belt, policymakers should consider mass vaccination campaigns among older children and adults to precede or accompany routine infant immunization. To guide policy decisions, there remains a substantial need for data on serotype-specific pneumococcal transmission patterns, pneumococcal pneumonia and invasive disease burden across age strata, and pneumonia and meningitis sequelae. A vaccine demonstration project [47] measuring impact on meningitis and pneumonia outcomes could provide this information as well as information on serotype-specific vaccine effectiveness, vaccine impact on meningitis, pneumonia, and carriage, and assessment of programmatic, logistic, and economic issues associated with vaccine introduction.

## List of abbreviations

GAVI: Global Alliance for Vaccines and Immunization; HIV: Human immunodeficiency virus; Sp: *Streptococcus pneumoniae*; WHO: World Health Organization.

## Competing interests

Bradford D. Gessner and Judith E. Mueller work for Agence de Médecine Préventive, which receives substantial financial support for all of its activities from sanofi pasteur, which is currently not involved in the production of pneumococcal conjugate vaccines. Additionally, both have received honoraria from Glaxo-Smith-Kline, a producer of pneumococcal conjugate vaccines.

## Authors' contributions

BDG conceived the analysis, developed the initial model, carried out all analysis, wrote the first draft of the manuscript, and read and approved the final manuscript.

JEM assisted with model development, provided substantial input into the interpretation of results, assisted in the preparation of the final manuscript, and read and approved the final manuscript.

SY provided substantial input into the interpretation of results, assisted in the preparation of the final manuscript, and read and approved the final manuscript.

## Pre-publication history

The pre-publication history for this paper can be accessed here:

http://www.biomedcentral.com/1471-2334/10/22/prepub

## Supplementary Material

Additional file 1 **Table S1**. Table S1: studies reporting the ratio of pneumococcal meningitis to bacteremia globally. Click here for file
