# Supplementary material for: African meningitis belt pneumococcal disease epidemiology indicates a need for an effective serotype 1 containing vaccine, including for older children and adults
Source: BMC Infect Dis. 2010 Feb 10;10:22. doi: 10.1186/1471-2334-10-22 (PMC2838886; doi:10.1186/1471-2334-10-22)
Supplement: Additional file 1 — Table S1. Table S1: studies reporting the ratio of pneumococcal meningitis to bacteremia globally. [file 1471-2334-10-22-S1.DOC]

Table S1. Studies reporting the ratio of *Streptococcus pneumoniae* (Sp) bacteremic pneumonia to meningitis throughout life.

|  |  |  | **Sp bacteremic pneumonia/meningitis ratio by age group: first number or range is age (in years), second is the ratio** | | | |
| --- | --- | --- | --- | --- | --- | --- |
| **Study site** | **Reference** | **Total annual invasive Sp incidence per 100,000 (cases)** | **Infancy/early childhood** | **Late childhood** | **Adult** | **Elderly** |
| **United States** |  | | | | | |
| Alaska: Non-Native | 16 | 16 (357) | <2: 3.0 | 2-19: 3.7 | 20-64: 13 | 65+: 23 |
| Metropolitan Atlanta, GA (2000-1) | 15 | 19 (504) | <18: 2.7 | | 18+: 8.8 | |
| Southern California | 17 | 13 (814) | <3: 1.7 | 3 to 64: 7.0 | | 65+: 32 |
| Charleston, SC | 18 | 19 (110) | <2: 3.5 | 2+: 49 | | |
| Dallas, TX | 19 | 22 (432) | <2: 1.8 | 2 to 29: 11 | 30 to 64: 14 | 65+: 15 |
| **Europe** |  | | | | | |
| France | 29 | 7.1 (214) | <16: 2.8 | | 16 to 44: 4.5  45 to 64: 9.5 | 65+: 15 |
| Spain | 28 | 11 (1927) | <2: 3.6 | 2-4: 11  5 to 14: 3.1  14 to 24: 3.3 | 25 to 34: 10  35 to 44: 5.7  45 to 64: 5.6 | 65+: 11 |
| Australia: non-Aboriginal | 20 | 16 (23) | <15: 4 | | 15+: Undefined (all pneumonia) | |
| **Indigenous populations** |  | | | | | |
| Alaska: Alaska Native | 16 | 77 (315) | <2: 3.8 | 2-19: 11 | 20-64: 6.8 | 65+: 8.3 |
| Arizona: Apache | 21 | 207 (138) | <5: 38 | 5-19: Undefined (all pneumonia) | 20+: 13 | |
| Australia: Aborigines | 20 | 222 (162) | <15: 4.6 | | 15+: 5.9 | |
